# Supplementary material for: The efficacy of dance for improving motor impairments, non-motor symptoms, and quality of life in Parkinson’s disease: A systematic review and meta-analysis
Source: PLoS One. 2020 Aug 5;15(8):e0236820. doi: 10.1371/journal.pone.0236820 (PMC7406058; doi:10.1371/journal.pone.0236820)
Supplement: S1 File — (DOCX) [file pone.0236820.s001.docx]

**Inclusion criteria**

1. Types of studies: (1) Peer reviewed randomized controlled trials
   1. Control groups included no intervention or a comparison group that practiced other forms of exercise, rehabilitation therapies, or social activities
   2. Randomized trials comparing two types of dance are also eligible
2. Patients diagnosed with Parkinson’s disease (as defined by authors of the studies)
   1. In order to compare across studies, the disease stage must have been reported and measured using the original or modified Hoehn and Yahr scale
   2. All ages and disease stages were included
3. Evaluated the outcome of a dance intervention for people with PD that lasted longer than one day
4. All settings in which dance intervention took place were considered, including community, hospital, rehabilitative center, or institution
5. Results reported at least one motor outcome, non-motor outcome, or measure of quality of life either self-reported or observed
