# Supplementary material for: The efficacy of dance for improving motor impairments, non-motor symptoms, and quality of life in Parkinson’s disease: A systematic review and meta-analysis
Source: PLoS One. 2020 Aug 5;15(8):e0236820. doi: 10.1371/journal.pone.0236820 (PMC7406058; doi:10.1371/journal.pone.0236820)
Supplement: S2 File — (DOCX) [file pone.0236820.s002.docx]

**Full Search Strategy**

Medline (Ovid) 1946 – March (Week 4) 2020

1. exp Parkinson Disease/

2. Parkinson*.mp

3. exp Dance Therapy/

4. exp Dancing/

5. danc*.mp

6. 1 or 2

7. 3 or 4 or 5

8. 6 and 7

Embase (Ovid) 1974 – March (Week 4) 2020

1. exp Parkinson Disease/

2. Parkinson*.mp

3. exp Dance Therapy/

4. exp Dancing/

5. danc*.mp

6. 1 or 2

7. 3 or 4 or 5

8. 6 and 7

PsycINFO (Ovid) 1806 to March (Week 4) 2020

1. exp Parkinson Disease/

2. Parkinson*.mp

3. exp Dance Therapy/

4. exp DANCE/

5. danc*.mp

6. 1 or 2

7. 3 or 4 or 5

8. 6 and 7

CINAHL Plus (EBSCOhost Research Databases) 1982 to March (Week 4) 2020

S1. (MH “Parkinson Disease”)

S2. parkinson*

S3. (MH “Dance Therapy”) OR (MH “Dancing”)

S4. danc*

S5. S1 OR S2

S6. S3 OR S4

S7. S5 AND S6

PubMed March (Week 4) 2020

#1 Parkinson disease

#2 Parkinson*

#3 Dance Therapy

#4 Dancing

#5 danc*

#6 (Parkinson disease) OR Parkinson*

#7 ((Dance Therapy) OR Dancing) OR danc*

#8 (((Parkinson disease) OR Parkinson*)) AND (((Dance Therapy) OR Dancing) OR danc*)
