# Supplementary material for: The efficacy of dance for improving motor impairments, non-motor symptoms, and quality of life in Parkinson’s disease: A systematic review and meta-analysis
Source: PLoS One. 2020 Aug 5;15(8):e0236820. doi: 10.1371/journal.pone.0236820 (PMC7406058; doi:10.1371/journal.pone.0236820)
Supplement: S3 File — (DOCX) [file pone.0236820.s003.docx]

**Risk of Bias Judgement Tables**

**Duncan & Earhart 2012**

| Bias | Authors’  Judgement | Justification |
| --- | --- | --- |
| Random sequence generation (selection bias) | Low risk | Participants randomized using online random number generator |
| Allocation concealment (selection bias) | Unclear risk | Concealment of allocation not described |
| Blinding of participants and personnel (performance bias) | High risk | It was not possible to blind participants and personnel due to the nature of dance interventions |
| Blinding of outcome assessment (detection bias) | Low risk | Assessors were blind to group assignment |
| Incomplete outcome data (attrition bias) | High risk | Dropouts > 10% (37% in control group, 50% in tango group) |
| Similarity at Baseline | Low risk | No significant differences at baseline for demographic measures or physical activity levels |
| Intention to Treat Analysis | Unclear risk | Intention-to-treat used with participants who completed baseline assessments + one other evaluation included (not all participants randomized were analyzed) |
| Eligibility Criteria | Low risk | Inclusion and exclusion criteria described |
| Consistency of Co-Interventions | Unclear risk | Monitoring of co-interventions (exercise, medication, etc.) during the course of the intervention is not described; participants tested OFF medication at all time points with time of day kept the same so unlikely that any effects were the result of pharmacological intervention |
| Comparability between trial arms | High risk | Unequal contact time between arms (2x per week for one year vs. no contact) |

**Duncan & Earhart 2014**

| Bias | Authors’  Judgement | Justification |
| --- | --- | --- |
| Random sequence generation (selection bias) | Unclear risk | Randomization method not described |
| Allocation concealment (selection bias) | Unclear risk | Concealment of allocation not described |
| Blinding of participants and personnel (performance bias) | High risk | It was not possible to blind participants and personnel due to the nature of dance interventions |
| Blinding of outcome assessment (detection bias) | Low risk | Trained, blinded physiotherapist completed assessments |
| Incomplete outcome data (attrition bias) | Low risk | No dropouts reported |
| Similarity at Baseline | Low risk | No significant differences between groups at baseline |
| Intention to Treat Analysis | Unclear risk | Method of analysis not described |
| Eligibility Criteria | Low risk | Inclusion and exclusion criteria described |
| Consistency of Co-Interventions | Unclear risk | Monitoring of co-interventions (exercise, medication, etc.) during the course of the intervention is not described; participants tested OFF medication at all time points so unlikely that any effects were the result of pharmacological intervention |
| Comparability between trial arms | Unclear risk | Unequal contact time between arms (2x per week for two years vs. no contact) |

**Foster et al., 2013**

| Bias | Authors’  Judgement | Justification |
| --- | --- | --- |
| Random sequence generation (selection bias) | Unclear risk | Randomization method not described |
| Allocation concealment (selection bias) | Unclear risk | Concealment of allocation not described |
| Blinding of participants and personnel (performance bias) | High risk | It was not possible to blind participants and personnel due to the nature of dance interventions |
| Blinding of outcome assessment (detection bias) | Low risk | Assessor was blinded to group allocation |
| Incomplete outcome data (attrition bias) | High risk | Dropouts > 10% (13% in control group, 19% in tango group) |
| Similarity at Baseline | Low risk | No significant differences between groups at baseline |
| Intention to Treat Analysis | Unclear risk | Intention-to-treat analysis used with participants who completed baseline and 3 month evaluation included (not all participants randomized analyzed) |
| Eligibility Criteria | Low risk | Inclusion and exclusion criteria described |
| Consistency of Co-Interventions | Unclear risk | Tested off medication; whether changes in medication was monitored during the course of the intervention is not described |
| Comparability between trial arms | High risk | Unequal contact time between groups (2x per week for one year vs. no contact) |

**Hackney et al., 2007**

| Bias | Authors’  Judgement | Justification |
| --- | --- | --- |
| Random sequence generation (selection bias) | Unclear risk | Randomization method not described |
| Allocation concealment (selection bias) | Unclear risk | Concealment of allocation not described |
| Blinding of participants and personnel (performance bias) | High risk | It was not possible to blind participants and personnel due to the nature of dance interventions |
| Blinding of outcome assessment (detection bias) | Low risk | Assessments were videotaped and data files coded for blinded ratings |
| Incomplete outcome data (attrition bias) | Low risk | No dropouts reported |
| Similarity at Baseline | Low risk | No significant differences between groups at baseline |
| Intention to Treat Analysis | Unclear risk | Method of analysis not described |
| Eligibility Criteria | Low risk | Inclusion criteria stated and definition of disease defined |
| Consistency of Co-Interventions | Low risk | Participants were instructed to continue with their ordinary exercise routines and did not engage in other dancing or group exercises classes during intervention; tested on medication at the same time pre and post |
| Comparability between trial arms | Low risk | Equal time and attention given to both groups (2x per week for 13 weeks) |

**Hackney & Earhart, 2009a**

| Bias | Authors’  Judgement | Justification |
| --- | --- | --- |
| Random sequence generation (selection bias) | High risk | Assigned by selecting condition out of a hat |
| Allocation concealment (selection bias) | Unclear risk | Concealment of allocation not described |
| Blinding of participants and personnel (performance bias) | High risk | It was not possible to blind participants and personnel due to the nature of dance interventions |
| Blinding of outcome assessment (detection bias) | Low risk | Assessments were video recorded for blinded ratings |
| Incomplete outcome data (attrition bias) | High risk | Dropouts >10% (22% in tango, 11% in waltz/foxtrot, 15% in control group) |
| Similarity at Baseline | Low risk | No significant differences between groups at baseline |
| Intention to Treat Analysis | Unclear risk | Per protocol analysis used |
| Eligibility Criteria | Low risk | Inclusion and exclusion criteria described |
| Consistency of Co-Interventions | Low risk | Participants were told to not make any changes to their exercise routines outside of class and excluded if medication changed; assessments took place at standardized time to avoid medication fluctuations |
| Comparability between trial arms | Low risk | Equal contact time given to both active groups (2x per week for 13 weeks for active groups vs. no contact control) |

**Hackney & Earhart, 2009b**

| Bias | Authors’  Judgement | Justification |
| --- | --- | --- |
| Random sequence generation (selection bias) | High risk | First author pulled a condition out of a hat |
| Allocation concealment (selection bias) | Unclear risk | Concealment of allocation not described |
| Blinding of participants and personnel (performance bias) | High risk | It was not possible to blind participants and personnel due to the nature of dance interventions |
| Blinding of outcome assessment (detection bias) | Unclear risk | Whether or not assessor was blinded is not stated |
| Incomplete outcome data (attrition bias) | High risk | Dropouts >10% (22% in tango, 11% in waltz/foxtrot, 24% in tai chi group, 15% in control group) |
| Similarity at Baseline | Low risk | No significant differences between groups at baseline |
| Intention to Treat Analysis | Unclear risk | Per protocol analysis used |
| Eligibility Criteria | Low risk | Inclusion and exclusion criteria described |
| Consistency of Co-Interventions | Low risk | Participants were excluded if they received alterations in their medication schedule and were instructed not to change their habitual exercise routines during the study; participants were tested ON at a standardized time to reduce medication-related fluctuations in performance |
| Comparability between trial arms | Low risk | Equal contact time given to all active groups (2x per week for 13 weeks for active groups vs. no contact control) |

**Hackney & Earhart, 2010**

| Bias | Authors’  Judgement | Justification |
| --- | --- | --- |
| Random sequence generation (selection bias) | High risk | First author pulled a condition out of a hat |
| Allocation concealment (selection bias) | Unclear risk | Concealment of allocation not described |
| Blinding of participants and personnel (performance bias) | High risk | It was not possible to blind participants and personnel due to the nature of dance interventions |
| Blinding of outcome assessment (detection bias) | Low risk | Assessments were video recorded and coded for blinded ratings |
| Incomplete outcome data (attrition bias) | High risk | Dropouts > 20% (37% in partner at follow up, 25% in non-partner at follow up) |
| Similarity at Baseline | Low risk | No significant differences between groups at baseline |
| Intention to Treat Analysis | Unclear risk | Intention-to-Treat analysis used with dropouts >10% |
| Eligibility Criteria | Low risk | Inclusion and exclusion criteria described |
| Consistency of Co-Interventions | Low risk | Participants instructed to continue with habitual exercise routine and medication state kept constant during assessments |
| Comparability between trial arms | Low risk | Equal contact time given to both groups (2x per week for 10 weeks) |

**Hulbert et al., 2017**

| Bias | Authors’  Judgement | Justification |
| --- | --- | --- |
| Random sequence generation (selection bias) | Unclear risk | Purposively sampled based on H&Y stage after being randomized by "remote telephone access randomization" |
| Allocation concealment (selection bias) | Unclear risk | Concealment of allocation not described |
| Blinding of participants and personnel (performance bias) | High risk | It was not possible to blind participants and personnel due to the nature of dance interventions |
| Blinding of outcome assessment (detection bias) | High risk | Assessor not blinded due to purposeful recruitment process |
| Incomplete outcome data (attrition bias) | High risk | Missing data >10% (1 drop out, 2 excluded at data collection in dance group) |
| Similarity at Baseline | Low risk | No significant differences between groups at baseline |
| Intention to Treat Analysis | Unclear risk | Per protocol analysis used |
| Eligibility Criteria | Low risk | Inclusion and exclusion criteria described |
| Consistency of Co-Interventions | Low risk | Medication kept constant during assessments and all participants continued with usual care |
| Comparability between trial arms | Unclear risk | Unequal contact time between groups (2x per week for 10 weeks vs. no contact) |

**Kunkle et al., 2017**

| Bias | Authors’  Judgement | Justification |
| --- | --- | --- |
| Random sequence generation (selection bias) | Unclear risk | Participants randomized in blocks; randomization method not stated |
| Allocation concealment (selection bias) | Unclear risk | Researcher received group allocation assignment by telephone from medical statistician but details are unclear |
| Blinding of participants and personnel (performance bias) | High risk | It was not possible to blind participants and personnel due to the nature of dance interventions |
| Blinding of outcome assessment (detection bias) | Low risk | Assessor was blinded to group assignment |
| Incomplete outcome data (attrition bias) | Unclear risk | Dropouts <10% overall but >10% in dance group (13% in dance, 0% in control) |
| Similarity at Baseline | Unclear risk | Trend toward longer diagnosis in control group |
| Intention to Treat Analysis | Unclear risk | Method of analysis not described |
| Eligibility Criteria | Low risk | Inclusion and exclusion criteria described |
| Consistency of Co-Interventions | Low risk | Medication kept constant at assessment sessions and participants in both groups continued with usual care outside of intervention |
| Comparability between trial arms | Unclear risk | Unequal contact time (2x per week for 10 weeks vs. no contact) with control group receiving vouchers to attend dance classes after intervention was complete to reduce chance of resentment or demoralization |

**Lee et al., 2018**

| Bias | Authors’  Judgement | Justification |
| --- | --- | --- |
| Random sequence generation (selection bias) | Low risk | Randomized using computer generated number sequence |
| Allocation concealment (selection bias) | Unclear risk | Concealment of allocation not described |
| Blinding of participants and personnel (performance bias) | High risk | It was not possible to blind participants and personnel due to the nature of dance interventions |
| Blinding of outcome assessment (detection bias) | Low risk | Assessors were blinded to group assignment |
| Incomplete outcome data (attrition bias) | High risk | Dropouts >10% (19% in control group, 12% in dance group) |
| Similarity at Baseline | Low risk | No significant differences between groups at baseline |
| Intention to Treat Analysis | Unclear risk | Intention-to-treat used with dropouts >10% |
| Eligibility Criteria | Low risk | Inclusion and exclusion criteria described |
| Consistency of Co-Interventions | Unclear risk | All patients continued to receive their routine pharmacological treatment and had no exercise therapy 3mos prior to study; whether participants were tested on medication and whether this was controlled is not stated |
| Comparability between trial arms | Unclear risk | Unequal contact time (2x per week for 8 weeks vs. no contact for 8 weeks plus intervention due to partial crossover design) |

**Michels et al., 2018**

| Bias | Authors’  Judgement | Justification |
| --- | --- | --- |
| Random sequence generation (selection bias) | Low risk | Randomized using computer block design |
| Allocation concealment (selection bias) | Unclear risk | Concealment of allocation not described |
| Blinding of participants and personnel (performance bias) | High risk | It was not possible to blind participants and personnel due to the nature of dance interventions |
| Blinding of outcome assessment (detection bias) | Low risk | Assessor was blinded to group assignment |
| Incomplete outcome data (attrition bias) | Low risk | All participants completed the study |
| Similarity at Baseline | High risk | Due to small sample, participants were not similar at baseline despite randomization |
| Intention to Treat Analysis | Low risk | Comparison of groups not carried out due to study being underpowered |
| Eligibility Criteria | Low risk | Inclusion and exclusion criteria described |
| Consistency of Co-Interventions | Low risk | Participants were on a stable PD medication regimen for at least one month prior and were excluded if they had participated in therapeutic dance interventions 3 months before the study or initiated any new PD treatments, medicines, or interventions |
| Comparability between trial arms | Low risk | Equal contact time between groups (1x per week for 10 weeks) |

**Rocha et al., 2018**

| Bias | Authors’  Judgement | Justification |
| --- | --- | --- |
| Random sequence generation (selection bias) | Low risk | Randomized using computer generated number sequences by a third party |
| Allocation concealment (selection bias) | Unclear risk | Concealment of allocation not described |
| Blinding of participants and personnel (performance bias) | High risk | It was not possible to blind participants and personnel due to the nature of dance interventions |
| Blinding of outcome assessment (detection bias) | Low risk | Assessor was blinded to group assignment |
| Incomplete outcome data (attrition bias) | High risk | Dropouts greater than 20% (30% in dance group, 27% in mixed dance group) |
| Similarity at Baseline | Low risk | No significant differences between groups at baseline |
| Intention to Treat Analysis | Unclear risk | Intention to treat used with participants who completed more than one class included in the analysis (3 only completed 30 minutes of the first class); not all participants randomized were analyzed with dropouts > 10% |
| Eligibility Criteria | Low risk | Inclusion and exclusion criteria described |
| Consistency of Co-Interventions | Low risk | All continued with usual activities and were tested at the same time of day during their medication cycle |
| Comparability between trial arms | Low risk | Equal contact time between groups (1x per week for 8 weeks) |

**Rios Romenets et al., 2015**

| Bias | Authors’  Judgement | Justification |
| --- | --- | --- |
| Random sequence generation (selection bias) | Unclear risk | Randomized using a random number generator; 3 participants sequentially assigned to experimental group after first 3 randomized to avoid excessive waiting times for the group intervention |
| Allocation concealment (selection bias) | Unclear risk | Concealment of allocation not described |
| Blinding of participants and personnel (performance bias) | High risk | It was not possible to blind participants and personnel due to the nature of dance interventions |
| Blinding of outcome assessment (detection bias) | High risk | Raters were not blinded |
| Incomplete outcome data (attrition bias) | High risk | Drop outs >10% (13% in control group and 39% in tango group; unclear percentage of "dropouts" vs "protocol violations" in tango group, i.e., some of the 39% may have completed post-testing without completing the required number of tango classes) |
| Similarity at Baseline | Unclear risk | Controls had significantly higher fall prevalence and exercised more regularly and for more hours per week at baseline |
| Intention to Treat Analysis | Unclear risk | Intention-to-treat used with dropouts >10% |
| Eligibility Criteria | Low risk | Inclusion and exclusion criteria described |
| Consistency of Co-Interventions | Unclear risk | Tango group instructed to continue with usual routine and not start any new exercise programs; control group given the option of continuing with regular exercise program or starting a new prescribed, self-directed program; how control group activity levels were monitored is not described; participant with medication change was included in ITT analysis |
| Comparability between trial arms | Unclear risk | Unequal contact time between groups (2x per week for 12 weeks vs. no contact) |

**Shanahan et al., 2017**

| Bias | Authors’  Judgement | Justification |
| --- | --- | --- |
| Random sequence generation (selection bias) | Low risk | Independent mediator blinded to the study hypothesis generated a random allocation sequence |
| Allocation concealment (selection bias) | Low risk | Concealment of allocation using sealed brown envelops |
| Blinding of participants and personnel (performance bias) | High risk | It was not possible to blind participants and personnel due to the nature of dance interventions |
| Blinding of outcome assessment (detection bias) | Low risk | Assessors were blinded to group assignment |
| Incomplete outcome data (attrition bias) | High risk | Dropouts > 20% (47% in dance group, 44% in control group) |
| Similarity at Baseline | Low risk | No significant differences between groups at baseline |
| Intention to Treat Analysis | Unclear risk | Per protocol analysis used |
| Eligibility Criteria | Low risk | Inclusion and exclusion criteria described |
| Consistency of Co-Interventions | Unclear risk | Participants continued their usual medication treatment, daily activities, and exercise habits during the study; no participants received additional therapies during the study and were excluded in the event of medication changes; whether medication was controlled for during assessments is not stated |
| Comparability between trial arms | Unclear risk | Unequal contact time between groups (1x per week for 10 weeks vs. no contact) |

**Solla et al., 2019**

| Bias | Authors’  Judgement | Justification |
| --- | --- | --- |
| Random sequence generation (selection bias) | Low risk | Random number program generator |
| Allocation concealment (selection bias) | Unclear risk | Concealment of allocation not described |
| Blinding of participants and personnel (performance bias) | High risk | It was not possible to blind participants and personnel due to the nature of dance interventions |
| Blinding of outcome assessment (detection bias) | Low risk | Assessors were blinded to group assignment |
| Incomplete outcome data (attrition bias) | Low risk | No drop outs, with one participant in the control group excluded from the analysis due to severe dyskinesia and freezing |
| Similarity at Baseline | Low risk | No significant differences between groups at baseline |
| Intention to Treat Analysis | Unclear risk | Method of analysis not described |
| Eligibility Criteria | Low risk | Inclusion and exclusion criteria described |
| Consistency of Co-Interventions | Low risk | Participants were instructed to continue with usual care and daily activities during the trial; assessments were carried out during ON phase; medication was monitored using a self-report measure and it is reported that there were no medication changes |
| Comparability between trial arms | Unclear risk | Unequal contact time between groups (2x per week for 12 weeks vs. no contact) |

**Volpe et al., 2013**

| Bias | Authors’  Judgement | Justification |
| --- | --- | --- |
| Random sequence generation (selection bias) | Low risk | Computer generated number sequences were generated by a third party; a blocked stratified randomization procedure was used based on H&Y |
| Allocation concealment (selection bias) | Low risk | Allocation was concealed by opaque envelopes |
| Blinding of participants and personnel (performance bias) | High risk | It was not possible to blind participants and personnel due to the nature of dance interventions |
| Blinding of outcome assessment (detection bias) | Low risk | Assessors were blinded to group assignment |
| Incomplete outcome data (attrition bias) | Low risk | No dropouts described |
| Similarity at Baseline | Low risk | No significant differences between groups at baseline |
| Intention to Treat Analysis | Unclear risk | Method of analysis not described |
| Eligibility Criteria | Low risk | Inclusion and exclusion criteria described |
| Consistency of Co-Interventions | Unclear risk | Medication was not controlled and testing did not always occur at peak dose in the medication cycle despite always being in the same hour |
| Comparability between trial arms | Unclear risk | Equal contact time between groups (1x per week for 6 months); however, Irish set dancing classes were delivered in a group setting while physiotherapy sessions were done individually |
