# Supplementary material for: The efficacy of dance for improving motor impairments, non-motor symptoms, and quality of life in Parkinson’s disease: A systematic review and meta-analysis
Source: PLoS One. 2020 Aug 5;15(8):e0236820. doi: 10.1371/journal.pone.0236820 (PMC7406058; doi:10.1371/journal.pone.0236820)
Supplement: Attachment — Submitted filename: PLOS review Feb 2020.docx [file pone.0236820.s005.docx]

Review: “The efficacy of dance for improving motor impairments, non-motor symptoms, and quality of life in Parkinson’s disease: A systematic review and meta-analysis.”

Summary of study

This manuscript presents a systematic review and meta-analysis of studies investigating the potential motor and non-motor benefits of dance for people with Parkinson’s disease (PD). The aims were to evaluate the evidence for the efficacy of dance in improving the above outcomes for people with PD, to assess the methodological quality of studies, and to identify future research directions.

While there are several recent reviews and meta-analyses on this topic, this is a fast-growing area of research and the present review considers a number of more recent studies in addition to the previous evidence.

The review focuses on randomised controlled trials, with 15 studies found to be eligible for inclusion, 8 of which were also included in a meta-analysis. The analysis indicated that dance can improve motor impairment in individuals with mild to moderate PD, but further research is needed to elucidate non-motor effects. The authors offer a number of suggestions for future research in this area, including multi-centre trials, investigation of underlying mechanisms, and effects in different groups of PD patients (advanced and young onset).

This is a clearly written, well-structured review of the current literature on dance for PD, which provides an update following previous reviews and meta-analyses on this topic.

Introduction

The authors refer to a number of existing systematic reviews and meta-analyses on dance for PD. They may also wish to note the following references:

*Lotzke, D., Ostermann, T., Bussing, A., 2015. Argentine tango in Parkinson disease – a systematic review and meta-analysis. BMC Neurol. 15. https://doi.org/10.1186/s12883-015-0484-0.*

*Aguiar, L.P.C., Da Rocha, P.A., Morris, M., 2016. Therapeutic dancing for Parkinson’s disease. Int. J. Gerontol. 10, 64–70. https://doi.org/10.1016/j.ijge.2016.02.002.*

Methods

Lines 138 and 154: Please correct “Cochran” to “Cochrane”.

Line 161: “In trials where two types of dance were compared to another active control or no intervention, the means and SDs of the two dance groups’ change scores were pooled” – could the authors please explain why this approach was taken, instead of separately examining the effects for each dance style?

Results

The authors do not consistently note the dance styles concerned when discussing findings (e.g., lines 401, 410) – it would be helpful to include this throughout.

Within the qualitative synthesis section, quality of life and activities of daily living are reported under the heading of “Non-motor symptoms” – it would be more appropriate to discuss these under separate headings as they can be impacted by a range of different symptoms.

Line 211: The authors note that only one study involved a programme led by a dance therapist – this is very interesting and it would be good to know what this is in contrast to; i.e., what was the training/background of the instructors leading the other programmes?

Table 1: The column “Analysis Method” is unclear – what does yes/no refer to here?

Line 252: “Six trials …controlled for medication during the course of the intervention” – please clarify in what way medication was controlled.

Line 333: Please correct “Kunkle” to “Kunkel” (also elsewhere in the manuscript).

Discussion

The discussion is comprehensive and highlights limitations in the existing literature, as well as providing suggestions for future research, such as the need to clarify underlying mechanisms of the benefits of dance in PD (e.g., which elements of dance might target which symptoms). However, it is quite a lengthy discussion and in parts repeats information that is also presented in the results – the authors could consider condensing some of this detail.

Line 644: This is a little unclear – do the authors mean “…reduced walking speed has been identified…”?

Line 662: The discussion of “mental imagery” is introduced in the section on motor outcomes – this would be better discussed in the non-motor section, where it is already considered in more detail.

Lines 687-692: The authors discuss the use of imagery within dance and the potential of dance to improve mental imagery skills. Please see the recent review by Bek et al. below, which discusses action representation mechanisms (motor imagery and action observation) in relation to dance for PD.

*Bek, J., Arakaki, A., Lawrence, A., Sullivan, M., Ganapathy, G., Poliakoff, E., 2020. Dance and Parkinson’s: A review and exploration of the role of cognitive representations of action. Neuroscience and Biobehavioral Reviews. 109. DOI: 10.1016/j.neubiorev.2019.12.023*

Lines 701-703: Please clarify that the study cited here focuses on PD.

Lines 732-734: The UPDRS-II scale (motor experiences of daily living) would be more appropriately discussed within the motor outcomes section rather than the QoL section where it is currently reported.

Conclusions and future directions

Line 817: “non-motor symptoms like cognition and depression” – this needs rephrasing as “cognition” is not a symptom.

Line 843: “…how this compares to other comparable interventions…” – this should be rephrased.
